# Detecting infected asymptomatic cases in a stochastic model for spread of Covid-19: the case of Argentina

**DOI:** 10.1038/s41598-021-89517-5

**Published:** 2021-05-11

**Authors:** N. L. Barreiro, T. Govezensky, P. G. Bolcatto, R. A. Barrio

**Affiliations:** 1 Instituto de Investigaciones Científicas y Técnicas para la Defensa (CITEDEF), Buenos Aires, 1603 Argentina; 2 Instituto de Invesitgaciones Biomédicas, Universidad Nacional Autónoma de México, 04510 Mexico, Mexico; 3 Instituto de Matemática Aplicada del Litoral (IMAL, CONICET/UNL), FHUC, Santa Fe, 3000 Argentina; 4 Instituto de Física, Universidad Nacional Autónoma de México, Apartado Postal 20-365, 04510 Mexico, Mexico

**Keywords:** Epidemiology, Statistical physics, thermodynamics and nonlinear dynamics

## Abstract

We have studied the dynamic evolution of the Covid-19 pandemic in Argentina. The marked heterogeneity in population density and the very extensive geography of the country becomes a challenge itself. Standard compartment models fail when they are implemented in the Argentina case. We extended a previous successful model to describe the geographical spread of the AH1N1 influenza epidemic of 2009 in two essential ways: we added a stochastic local mobility mechanism, and we introduced a new compartment in order to take into account the isolation of infected asymptomatic detected people. Two fundamental parameters drive the dynamics: the time elapsed between contagious and isolation of infected individuals ($\alpha$) and the ratio of people isolated over the total infected ones (*p*). The evolution is more sensitive to the $p-$parameter. The model not only reproduces the real data but also predicts the second wave before the former vanishes. This effect is intrinsic of extensive countries with heterogeneous population density and interconnection.The model presented has proven to be a reliable predictor of the effects of public policies as, for instance, the unavoidable vaccination campaigns starting at present in the world an particularly in Argentina.

## Introduction

The pandemic of 2020 has changed life in many respects. The scientific community was not indifferent to the urgency to find strategies to face up with this global disease. More than 23,500 articles published containing the word COVID-19 in the last year account for this fact. All the issues related with this phenomenon have been covered. There are studies on the medical and biological aspects of the virus, the mechanisms of contagion, the strategies to avoid the spread of the disease, the comprehension of the dynamical evolution of contagion, etc. Even recommendations to health authorities to prevent infection as to keep a respectable distance for others, the use of masks, washing hands profusely and frequently, and compulsory test and tracking techniques, measurements of air quality, between others^1^.

When a new virus emerges, and there is not effective treatment or vaccine yet, non-pharmacological interventions (NPIs) constitute the main response option for mitigating the effects of the pandemic. Assumed as an extreme measurement, total confinement (or quarantine) has been applied since the 14th century^2^. It is obviously effective because no personal contact means no infection spread, but, in the modern societies, this NPI is not feasible for a long period of time. Nevertheless, its effectiveness increases when applied early in the pandemic and in combination with other NPIs^3,4^. A recent action that has proved useful is to detect not only infected, but also asymptomatic people by random testing and isolate the ones that come out positive^5^.

Researches based on observational data and mathematical models are an irreplaceable tool in order to help to identify effective NPIs. Active searching for infected people who are asymptomatic or present mild symptoms and subsequent isolation was not included in the above mentioned researches, probably because few countries had implemented it. For SARS-CoV-2 a great proportion of infected people are asymptomatic or present mild symptoms^6,7^, however, they are mainly not detected. Consequently they still remain infectious and becomes transmission vector of the disease^8^. More research is needed to further assess the effect of this kind of combined procedure of NPIs.

Several compartmental epidemiological models have been expanded to include quarantined individuals^9–12^. The classical approaches assume general homogeneous populations and equally probable interactions among people. However, when modeling pandemics, demographic heterogeneity and people mobility could be key elements to be considered. In particular, human mobility is strongly affected by governments’ policies and it is almost imperative to include this feature in order to render meaningful simulations comparable to the real data. Because of this, several models have been proposed to include the actual geographic spread using real data or time-dependent parameters to simulate people’s mobility^13–20^.

Here we propose a model in which the noisy interactions of human society and the inhomogeneities of geographical spread are taken into account. We use an extension of a model proposed in Ref.^20^ to predict the influence of these measures of detection and subsequent isolation. The original model has been extremely successful in predicting the behavior of COVID-19 in various countries, as different in all respects as Mexico, Finland, and Iceland^21^. The dynamic works in two scales: on the one side a micro or local one in which the disease spread follow a (almost) standard compartmental evolution. It contains the biology-related parameters of the disease. On the other side, a macro or long range dynamics, which describes the geographical interconnection in a given region or country. However, the implementation as in^21^ was surprisingly inaccurate for the Argentina case. To understand this singular behavior we have to give more versatility to the model by incorporating the influence of detecting and isolating infected asymptomatic people as well as to account for local stochastic mobility.

In the next Section we describe the model in detail, then we explain how it is applied to the Argentina case, and then present some results from numerical calculations for the period from March to December of 2020. Finally we conclude with some important remarks.

## Theoretical model

The approach is based on a collection of SEIR models acting in cells distributed along (and filling) the whole geography of the country. The network is weighted by the population density of each cell. Connections between cells are realized by the national ground and/or air roads. This approach has the advantage that the parameters proper to the disease (only in the SEIR part of the model) are separated from the ones related to spreading infections between people, which ultimately translate into mobility quantities between cells.

### Local dynamics: SEIQR stochastic model (mycrodinamics)

**Figure 1:**
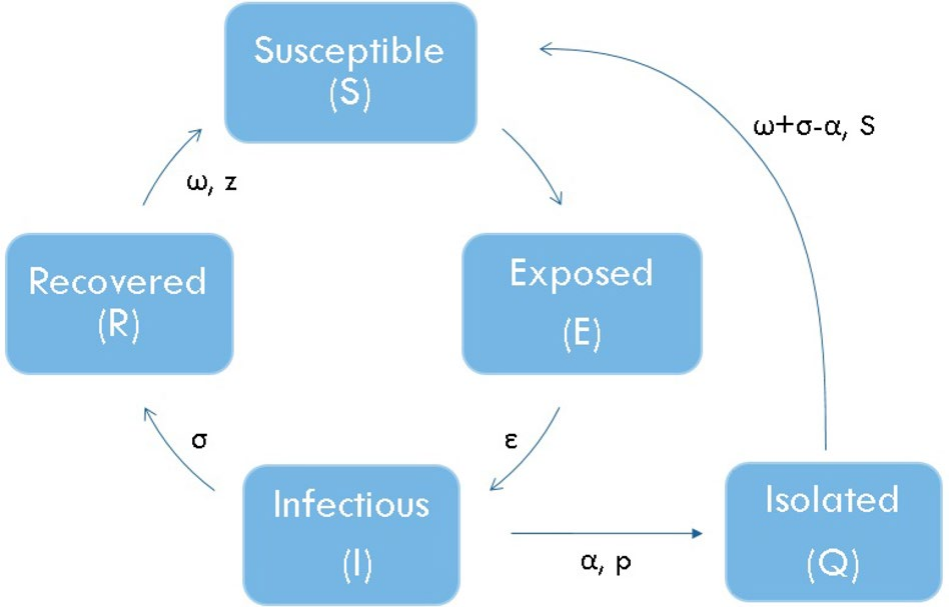
Compartment scheme of a SEIQR model. $\epsilon$, $\alpha$, $\sigma$ and $\omega$ are the latency, isolation, infectiousness and immunity periods, *p* is the portion of infected people which is isolated and *z* is the survival parameter.

The SEIR model of Ref.^20^ was converted into a SEIQR stochastic model in order to analyse the influence of detection and isolation of infected people. This was done by adding a quarantine compartment, *Q*. Figure 1 shows a diagram of the five-compartmental model: susceptible individual (*S*), exposed but not infectious yet (*E*), infectious (*I*), isolated in quarantine (*Q*), and a last compartment named recovered (*R*) but including the deaths also. Some parameters drive the link between compartments: incubation period ($\epsilon$), infectiousness period ($\sigma$ ) and immunity period ($\omega$) are parameters depending on the specific disease studied and host’s immune response. After the immunity period, people could be again susceptible to the disease according to the survival parameter *z*. Two new parameters are added to the model: $\alpha$—the time lapsed from infection to detection (and isolation) of infected individuals, and *p*—the proportion of infected people detected and put in quarantine, either symptomatic or asymptomatic.

Demography is added using a constant mortality rate $\mu$=1/*L*, where *L* is life expectancy. The total population $N=S+E+I+Q+R$ depends only on the survival parameter *z*; at time zero $S=N$. In order to keep *N* fixed, the birth rate is considered a constant ($\mu \cdot N$) and all the newborns are included in the susceptible compartment. Since we assume that the disease is spread due to daily contacts, the dynamical evolution of the model should be given in discrete steps of one day, which is the time unit of all the delay parameters. All time parameters are assumed to be constant along the simulations.

With all these assumptions, the SEIQR model -acting in each cell of the geographical region of interest- could be written as discrete mathematical map with five variables,

1 $$\begin{aligned} S_{t+1}= & {} (1-\mu )~(S_{t}- G_{t}+ z~(1-\mu )^{\epsilon +\sigma + \omega } G_{t-1-\epsilon -\sigma - \omega }) + N \cdot \mu \end{aligned}$$

2 $$\begin{aligned} E_{t+1}= & {} (1-\mu ) (E_{t}+ G_{t} - (1-\mu )^{\epsilon } G(t-1-\epsilon ))\end{aligned}$$

3 $$\begin{aligned} I_{t+1}= & {} (1-\mu )~(I_{t}+ (1-\mu )^{\epsilon } G_{t-1-\epsilon }- (1-p)~(1-\mu )^{\epsilon +\sigma } G_{t-1-\epsilon -\sigma }-p~(1-\mu )^{\epsilon +\alpha } G_{t-1-\epsilon -\alpha })\end{aligned}$$

4 $$\begin{aligned} Q_{t+1}= & {} (1-\mu )~(Q_{t}+p~(1-\mu )^{\epsilon +\alpha } G_{t-1-\epsilon -\alpha }-p~(1-\mu )^{\epsilon +\sigma + \omega } G_{t-1-\epsilon -\sigma - \omega } )\end{aligned}$$

5 $$\begin{aligned} R_{t+1}= & {} (1-\mu )~(R_{t}+(1-p)~(1-\mu )^{\epsilon +\sigma } G_{t-1-\epsilon -\sigma }- (1-p)~(1-\mu )^{\epsilon +\sigma + \omega } G_{t-1-\epsilon -\sigma - \omega }) \end{aligned}$$

where $G_t$ is the incidence function evaluated at time *t*. Assuming a homogeneously mixed population within a cell, the probability of getting infected is calculated using the Poisson probability distribution; then the incidence rate is given by: $G_t = S_t (1- e^{- \beta I_t}),$ where $\beta$ is a transmission parameter that characterizes the intrinsic behavior of the disease and it is a dimensionless constant We assume $G_t=0$ for $t < 1$.

While this system of equations is deterministic, people do not move only to fixed places following daily routine activities, but also may go to nearby unpredictable places. Thus, a random mobility within a cell should also be considered^22^. We denote as *local mobility*. This is done by adding a threshold parameter $\nu _L<1$ that accounts for people’s short distance mobility. Each day *t*, in every geographical cell with coordinates (*i*; *j*), we compare a random number (*r*, with uniform probability distribution between 0 and 1) with this threshold parameter. If $\nu _L<r$ the systems keep its normal evolution but, if $\nu _L>r$ the mobility was lower enough so that the epidemic is not able to evolve. In this case, there are not new infected people during this day in the cell under consideration.

### Geographical disease spreading (macrodynamics)

A realistic model of epidemics must include its geographical spread in big regions or counties with heterogeneous population densities. For this purpose, the map of the country under study is divided in a two dimensional grid of squares of size of a few $\hbox {km}^2$. For each cell of coordinates (*i*; *j*), the actual population density $\rho (i; j)$ is known. Within each cell population is normalized to $N=1$, local dynamics is simulated by a SEIQR stochastic model using the incidence function weighed by its population density:

6 $$\begin{aligned} G_t(i,j) = S_t(i,j) \rho (i,j) (1- e^{- \beta I_t(i,j)}) , \end{aligned}$$

To consider the spread among first neighbor regions we use a Metropolis Monte-Carlo algorithm. For each square in the grid, if $I_t(i,j)$ $\le$ $\eta$ and $\nu _n <r$, there is propagation of the disease to a neighbor cell. The value $\eta$ is related to the infectiousness of the disease and $\nu _n$ varies between 0 and 1 and accounts for the mobility between neighbors. *r* is a random number given by a uniform distribution between 0 and 1. To start the disease in a new cell of coordinates $(i,j+1)$, which is one first neighbor of the cell (*i*, *j*), the initial conditions are given by $I_t(i,j+1)=\eta$ and $S_t(i,j+1)=1-\eta$. The disease can also be spread randomly to distant regions because of people traveling between connected cities. Another Metropolis Monte-Carlo algorithm is used for long distance new infections, either by road or by air. It is more likely that bigger cities are infected first because they are more populated and connected. Because of this, the long distance mobility parameter $\nu _a$ is weighed by the normalized densities of both, origin and destiny cells. In this case propagation occurs between a cell already infected ($I_t(i,j)$ $\le$ $\eta$) and a cell connected to the first one by air, trains or national routes. If $\nu _a \rho (i,j) \rho (m,n) <r$, (with *r* a random number from a uniform probability distribution between 0 and 1) the cell at the new coordinates (*m*, *n*) starts the disease with initial conditions $I_t(m,n)=\eta$ and $S_t(m,n)=1-\eta$.

Finally, since people occasionally move in an apparent random way, it is possible to find people travelling to distant cities or even isolated towns with lower population densities. This is accounted for by the noise parameter *KT* representing the “kinetic energy” of the system. In this case a new Monte-Carlo algorithm is applied. For each cell with coordinates (*i*, *j*) with $\rho (i,j)> T$, (with T a normalized population density threshold), if $e^{-1/KT}<r$ (*r* a random number with uniform probability distribution between 0 and 1), then the disease will start at the cell (*i*, *j*) with initial conditions $I_t(i,j)=\eta$ and $S_t(i,j)=1-\eta$.

In this model $\beta$ does not depend on $\rho$ and/or on mobility of people as in traditional SEIR models. $\beta$ is considered constant throughout the pandemic, and mobility parameters can be used to reflect measures applied by different governments trying to control the pandemic.

Another advantage of this model is that the detected and reported individuals are clearly separated from non-reported ones, the model traces both groups. This is an important point in the case of COVID-19 where there are so many asymptomatic and mildly symptomatic people which dramatically impact on the spread of the viruses.

## Results

### Application of the model to COVID-19 in Argentina

**Figure 2:**
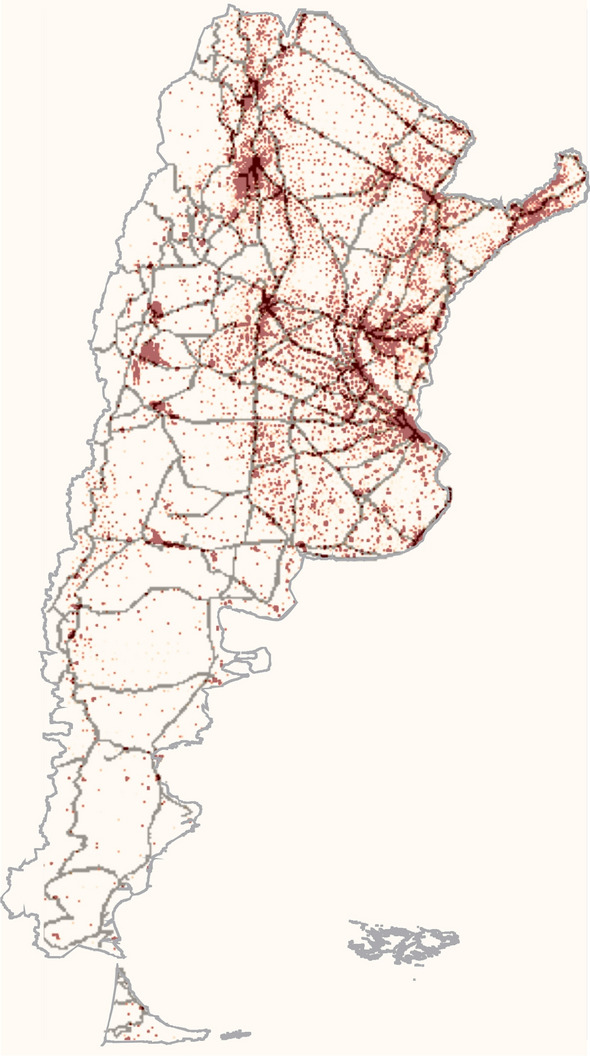
Density map and distribution of routes used in the model. The information to create the maps was provided by the IGN. Each pixel corresponds to a 7 km x 7 km parcel.

In order to apply these ideas to the case of Argentina we have divided the continental part of the country’s territory in a grid of around 67000 squares of 7 km $\times$ 7 km. The total population inside of each parcel was assigned from the data provided by the National Geographic Institute of Argentina (IGN). The interconnection between cities by commercial flights was canceled by public policies since the early days of the pandemic. Consequently, only land connections are possible. Therefore, we allow traveling (both, short and large distances) across the network of roads and routes also provided by the IGN (see figure 2).

### Fitting parameters to Argentina

For the process to fit parameters, it is important to remark that in this model the disease parameters are well separated from those that account for the social distancing and mobility. Since there is not much information about latency, infectiousness and immune periods of COVID-19, and this values could vary significantly from person to person, one can fit this values to the actual data within upper and lower thresholds found in the literature^23^.

The first confirmed case of COVID-19 in Argentina was a person coming from abroad on March 3th, 2020. And, in general, the disease evolution during the first 20 days was because of infected people coming from Europe and USA and not because of community spread. On March 20th a strict lock-down started all around the country and mobility was drastically reduced. Because of this, disease parameters cannot be fitted straight from Argentina data. In this sense we decided to use the same parameters as in Mexico, Iceland and Finland models^21^: $\epsilon =1$, $\sigma =14$ and $\beta =0.91$. $\omega$ was conservatively chosen as 140 days because the center for disease control and prevention (CDC) states that there are no reports of people being reinfected within 5 months of first infection^23^ . $\sigma$ is set to the quarantine standard time used in several countries (Argentina among them). A resume of the main parameters and the values used can be found in Table 1.

**Table 1:** Model parameters.

Parameter	Description	Value	Reference
$\epsilon$	Latency period	1	^21,31^^a^
$\sigma$	Infectiousness period	14	^21,32^^a^
$\omega$	Immunity period	140	^23,33^
$\beta$	Transmission parameter	0.91	^21^^b^
$\eta$	Triggers the dynamic in a new cell. Corresponds to start the disease in a city with at least 10 infected people	$10^{-5}$	Estimated from geographical data.
*z*	Survival parameter	0.9973	Case fatality rate 2.7$\%$ obtained from data^28^. Fatality rate depends on the amount of tested cases (10% of infected for $p=0.1$)
*KT*	Noise parameter	0.1	Estimated from NPIs

^a^In the case of SARS-Cov-2, infectiousness starts before the onset of symptomatic period^31,32,34,35^. Therefore, in terms of the model, $\epsilon$ is shorter than the non-symptomatic period (1–6 days^31,36^) and $\sigma$ is larger so that compartment *E* includes noninfectious people, and compartment *I* includes all the infectious ones. Since presymptomatic infections occur up to 4 days^32^ before symptoms onset, and most COVID-19 ill people can have replicable viruses 10 or more days after that^33^, we estimate $\epsilon$ and $\sigma$ as showed above.

^b^$\beta$ was obtained by fitting the model with the mentioned $\epsilon$ and $\sigma$ values in Ref.^21^.

At present, there are scarcely any available studies in Argentina to assure how many infected people is detected and put in quarantine (quantified in our model by the $p-$ parameter). One particular study in urban slum dwellers of Buenos Aires City suggests that only 10$\%$ of the actually infected peoples was PCR tested and nearly 90$\%$ were asymptomatic cases^24^. Patients are tested only when they present two or more concurrent symptoms and, in the case of a positive result, all of the close contacts are isolated independently of new tests. Since some studies suggest that most COVID-19 patients have mild symptoms or are asymptomatic^25–27^, we estimate that tested and isolated cases in Argentina are between 10$\%$ and 20$\%$ of the actual number of infected people, i.e. $0.1 \le p \le 0.2$. These values are similar in order of magnitude to those found in Spain and France^6,8^. To estimate $\alpha$ we use the information provided by the Health Ministry of Argentina for each patient^28^ . From this data, we found that 80$\%$ of the people start the isolation between 3 and 7 days after they get infectious although mostly at 5 days. Since this is a mean value model, we decided to use 5 as a good estimation. Conservatively, we choose the values $p=0.1$ and $\alpha =5$ to study the evolution of the pandemic in Argentina.

For simplicity, mobility parameters are regarded equal $\nu _a$=$\nu _l$=$\nu _n$=$\nu$. Consequently when they change, they do it at the same time in the same way. Since mobility was drastically reduced during the first stages of the pandemic, the noise was considered very low taking a value $KT=0.1$. We take four intervention times to account for the changes in the stringency of the government measures in the period March–December 2020. This intervention dates were fitted with a delay of around 7–8 days with the real implementation dates because we noticed that this is, in average, the time taken by a measure to impact in the growth rate of the pandemic. The government measures are described in Table 2. As it is shown in the table, five values of $\nu$ were fitted to daily cases for the different intervention periods. The fitting was done until day 180 verifying that real data were included within one standard deviation from the mean value taken from 100 simulations. A two-sample Kolmogorov-Smirnov test was also applied to verify the goodness of the fit.

**Table 2:** Mobility according to government measures.

Period (days)	$\nu$	Government measures
1 to 22	0.33	Schools and mass attendance events were closed and people were asked to stay at home
23 to 78	0.135	Strict lock-down in all the territory
79 to 101	0.185	Banks and other businesses were allowed
102 to 165	0.225	After a massive strike the pandemic evolution raised consistently
		The lock down started to be lifted
166 to present	0.358	Some restrictions were gradually lifted. The pandemic spread over all the country

### Comparison with reported data

**Figure 3:**
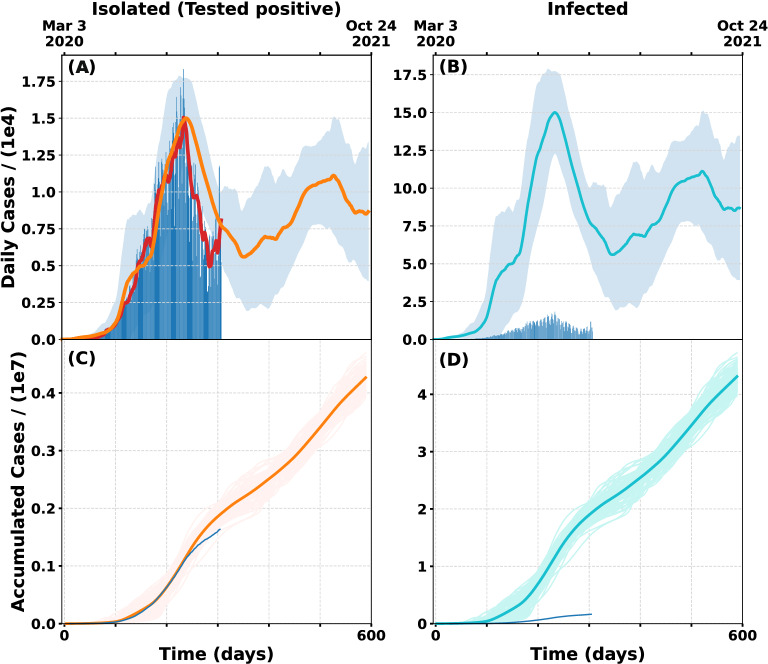
Geographical spread stochastic SEIQR Model fitted to Argentine data for $p=0.1$ and $\alpha =5$. (**A**) Daily isolated cases of infected people who were discovered, tested positive for COVID and isolated (orange lines) compared to official data (blue bars). The light blue shaded area corresponds to one standard deviation from the mean. (**B**) Daily infected cases in turquoise compared with official data (blue bars). Notice that infected are 10 times higher than the actual official data. (**C**) Time evolution of accumulated isolated cases obtained as the mean value (orange line) of 100 model runs. Light orange curves are the 100 individual numerical runs. The blue curve corresponds to official accumulated cases. (**D**) Time evolution of accumulated infected cases obtained as the mean value (turquoise line) of 100 model runs (lighter curves).

In Fig. 3 we show the result of this model obtained from adding the newly isolated people from all the cells in the grid scaled by each region’s own population. This plot is obtained by averaging 100 model runs. We should keep in mind that most governments are not able to detect all the actual COVID-19 cases but a fraction *p* and that only the confirmed and isolated people should be compared with the data provided by official sources.

In order to understand Fig. 3, three points should be kept in mind. First one is that the mobility parameter from day 166 onward was fixed to 0.358. This means that this prediction will be adequate as long as the people keep respecting social distancing measures. It should be regarded that if all the mobility and group meeting restrictions are lifted the evolution will be different. The second topic to consider is the appearance of a second pandemic wave. This wave is strongly related with the immunity period which was fixed at 140 days. Since there is not enough data available at this time it is possible that this second wave appears a few weeks earlier or later. Obviously, this dynamic will be dramatically different if a campaign of massive vaccination occurs.

The third issue to notice is that, as it is expected, the infected are 10 times higher than the isolated as it can be observed by comparing figures (A) and (B) or (C) and (D). At the current transmission rate, this implies that by the end of 2021 the pandemic will have infected a number of people equivalent to the country’s population. If we compute fatalities as the 0.027% of the infected, we can predict that there will be 116.000 deaths by the beginning of 2022.

**Figure 4:**
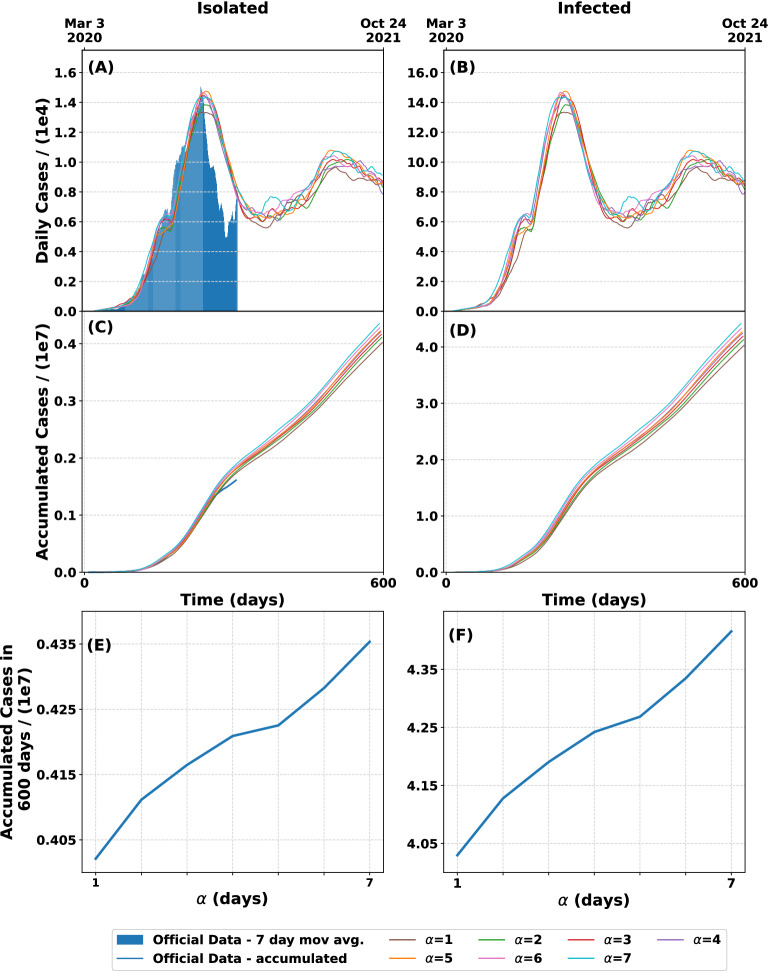
Model prediction according to different values of the parameter $\alpha$, the period of time between infection and isolation. For each curve we did 100 runs of the model. The value *p* is fixed to 0.1 and the mobility is the one fitted for $\alpha =5$. (**A**) Daily discovered (isolated) cases for different values of $\alpha$. (**B**) Daily actually infected cases for different values of $\alpha$. (**C**) Accumulated discovered (isolated) cases as function of time. The blue curve represents the temporal evolution of the actual accumulated cases obtained from official data. (**D**) Accumulated infected cases as function of time. (**E**) Accumulated isolated cases after 600 days as a function of $\alpha$. (**F**) Accumulated infected cases after 600 days as a function of $\alpha$. The longer the time to discover and isolate new infected cases, the greater the number of patients expected and, therefore, the greater the number of isolated people.

In order to study how the *p* and $\alpha$ parameters affect the evolution of the pandemic, we analyzed different combinations of them and fixing the values of $\nu$ as in the Table 2. Firstly, we study the model by varying $\alpha$. Figure 4 shows the curves obtained from 100 runs of the model for $p=0.1$ and different values of $\alpha$. As it is clear from the figure, the early discovery of a case reduces the height and the width of the peak in (A) and (B) but not in a significant way. This can be seen clearly in the curves (E) and (F) were the accumulated isolated and infected cases in 600 days are shown. The expected difference between early and late case discovery for $p = 0.1$ is around 8%, which is within the spread of the model. The value taken by $\alpha$ could become more significant for bigger *p* but, as we have seen before, the fraction of discovered and isolated people is small in most countries.

**Figure 5:**
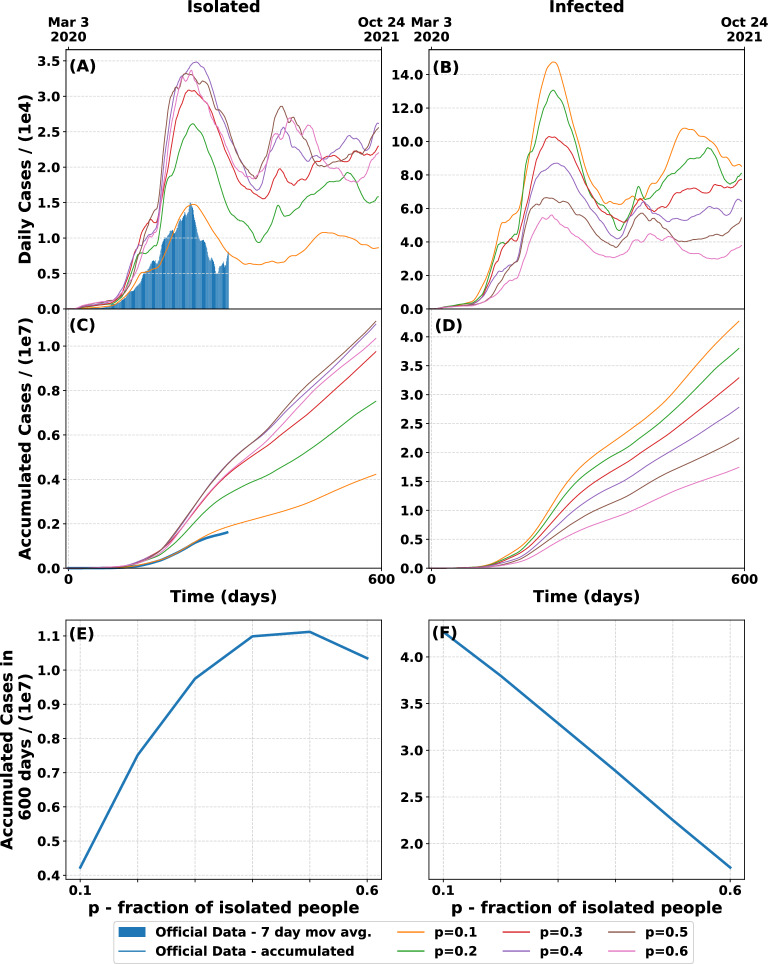
Prediction of geographical spread SEIQRS model according to different values of *p*. For each curve we did 100 runs of the model. The value $\alpha$ is fixed in 5 days and the mobility is the one fitted for $p=0.1$ (**A**) Daily discovered (isolated) cases for different values of *p* compared with official data. (**B**) Daily actually infected cases. (**C**) Time evolution of accumulated isolated cases for different values of *p*. (**D**) Time evolution of accumulated infected cases for different values of *p*. (**E**) Accumulated isolated cases after 600 days as a function of the fraction of isolated infected people. (**F**) Accumulated infected cases after 600 days as function of the value *p*. In this case, the larger is the value of *p* the smaller is the amount of accumulated infected after 600 days.

Next we studied the variation of the fraction of isolated people, *p*. The results can be seen in Fig. 5. It is clear from the figures (C) and (E) that the accumulated isolated are smaller for lower values of *p*. This result is reasonable since the higher the fraction of discovered infected people, the smaller is the population that continues to infect others reducing the spread of the pandemic. This shows that the implementation of an efficient COVID-19 tracking and testing program could be of great significance to control its evolution.

Figure 5D and F show the number of accumulated infected cases. In this case, as the number of discovered cases raises, the infected population decreases leaving a smaller pool of people with the virus to be found. Therefore, once that more than 40% of those infected are discovered, a decrease in the number of isolated cases is observed as a consequence of the reduction in the total diseased population. It is interesting to notice that, for the same parameters and mobility, we found that the total accumulated infected cases in 600 days is 57% smaller for $p = 0.6$ than for $p = 0.1$. This would imply a reduction in the expected fatalities to less than 50.000.

## Discussion and conclusions

In this work we have proposed a novel model to study the influence of geographical and sociological conditions on the spread of the virus SARS-CoV2, causing the COVID-19 pandemic in Argentina.

We introduce two fundamental parameters: $\alpha$, the time elapsed between contagious and isolation of infected individuals, and *p*, the ratio of people isolated over the total infected ones. The results show clearly that the detection and consequent isolation of infected people are crucial to the dynamics of the virus propagation ($p-$sensitivity) while the time lasted between infection and isolation is not a relevant issue ($\alpha -$insensitivity).

We also introduced local mobility into the model to account for random social behavior within each cell. This allowed a better prediction of the pandemic evolution and enabled the appearance of new features that were actually observed in real data (uneven slowed-down pandemic growth).

The model also predicts new waves which are dependent on the immunity time parameter ($\omega$) and are modulated by the NPIs. This feature was already observed in some European countries where “stay at home” policies were taken: a second wave appeared as soon as restriction were lifted and reinfection was possible.

**Figure 6:**
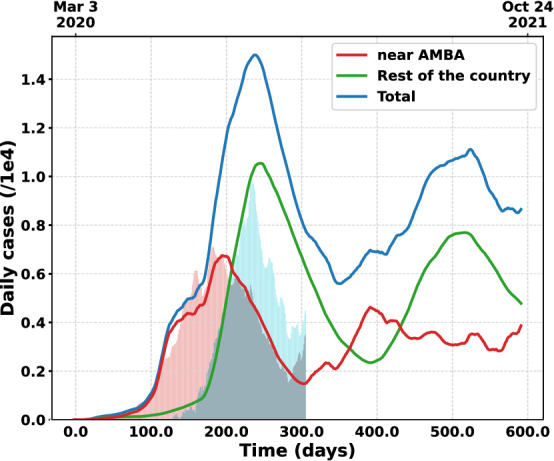
Prediction of the geographical evolution of the pandemic for $p = 0.1$, $\alpha = 5$ and the mobility of the Table 2. Three curves are shown: daily cases in Argentina, in the near AMBA region (city of Buenos Ares and the closest urban conglomerates, not the entire metropolitan area) and in the rest of the country. The bars shaded in red and green represent the official daily cases obtained from the Ministry of Health of Argentina^28^ for both geographic areas. The model shows a 50-day delay between the peak in the near-AMBA region and the rest of the country. This fact prevents the complete suppression of the disease.

Moreover, the model shows an interesting behavior in countries with a wide geographic span and high mobility. Particularly, it accurately describes the geographical spread of the pandemic in the Argentinean territory. From all the figures it can be clearly seen that the model predicts the appearance of a second wave before the end of the first one. This implies that , without any government measures as mobility reductions, effective infectious tracking or vaccines, the disease is not expected to disappear by itself. This is a direct consequence of the geographical extension of the territory and the stochasticity of the model. When the pandemic starts in a certain region of the country it also diminishes in another area at the same time (see Fig. 6). In this way, the disease oscillates between different regions delayed in time. Consequently, it never completely stops or vanishes. For instance, Fig. 6 show the pandemic evolution divided in two geographical areas: The main part of metropolitan area of Buenos Aires (AMBA) and the rest of the country. The illness started mainly in the AMBA region, which concentrates almost 33% of the population of Argentina. After several months of evolution, the pandemic moved to other important urban areas of the country as Córdoba, Santa Fe, Río Negro, Mendoza, Chaco, etc., and started diminishing in the AMBA region. The model predicts a $\sim$50-day elapsed between maxima on both areas which is very close to that actually seen in the official data. The great mobility between these areas will eventually create a new peak in the AMBA region once the immunity period is finished for most of its population and some restrictions are lifted.

This kind of geographic behaviour can also be seen in countries like USA, Brazil and Mexico because they have big territories with several important urban areas. As an example, USA had its first cases peaks in the North east states during April and then the pandemic moved to south western states where the cases peaks where on July. Because of this it never experienced an overall decrease in the amount of cases as in most European countries and, as a consequence, they are facing the second wave in states like New York without reaching the end of the first wave in other states.

As a conclusion,for large territory countries, the new daily cases are expected to come from different regions at different moments as the pandemic evolves. This creates unsynchronized oscillations of the daily case curves for different areas and prevents the disease from being completely eradicated. In order to stop this kind of behavior, a better control of the mobility between distant regions should be adopted. Testing and quarantine policies already embraced in some countries could prevent the appearance of new infectious foci.

The model described in this paper accurately accounts for the pandemic evolution in Argentina and in other countries as well^21^. Nevertheless, it should be noted that this prediction is limited by several assumptions. For instance long-term predictions are restricted by the available information on the mobility parameters. Their value is governed by social compliance with government restrictions and recommendations, and they can change rapidly if interventions are lifted or tightened. New government policies and the obedience of ordinary people to the recommendations cannot be predicted accurately in the long-term. This reduces the predictive power of the model. Additionally, when this model was developed, most parameters were unknown and were estimated from the available information and by fitting to real data. For instance, we assumed $\omega =140$. If this value changes new pandemic waves could appear before or after it was predicted. Other limitation is that we are not taking into account new strains with different properties. SARS-CoV-2 variants exhibit an increase in the transmission rate and in the infectiousness period, giving place to a growth in daily new cases in some countries^29^. Finally, reduction of the daily cases because of vaccination is neither considered in this model.

As final remarks, we want to emphasize the importance of tracking and isolating infected people. In this sense, countries as Iceland and South Korea have shown the effectiveness of these methods to reduce the pandemic spread^21,30^. On the other hand, cultural habits and social behavior have shown to be important factors as well. The increase in the number of cases because of the mobility growth was clearly demonstrated in the fitted $\nu$ parameters. With this in mind, social distancing measures and case tracking are two key factors to contain the pandemic evolution. Furthermore, as this model shows robustness as a global predictor in a very extensive and heterogeneously connected country like Argentina, we are confident that it gives strong support to analyze vaccination strategies for the future mitigation of the disease.

## Data Availability

The information to create the “daily cases” dataset used during the current study is available in the repository of the Health Ministry of Argentina. http://datos.salud.gob.ar/dataset/covid-19-casos-registrados-en-la-republica-argentina. Datasets generated during this study will be available from corresponding author upon request.

## Code availability

Python codes used during the current study are available from the corresponding author upon request.
